# Supplementary material for: The causal relationship between the human gut microbiota and pyogenic arthritis: a Mendelian randomization study
Source: Front Cell Infect Microbiol. 2024 Nov 26;14:1452480. doi: 10.3389/fcimb.2024.1452480 (PMC11629706; doi:10.3389/fcimb.2024.1452480)
Supplement: Supplementary file 1 [file DataSheet1.zip › mendelian test/ebi-a-GCST90027527.csv_forest.pdf]

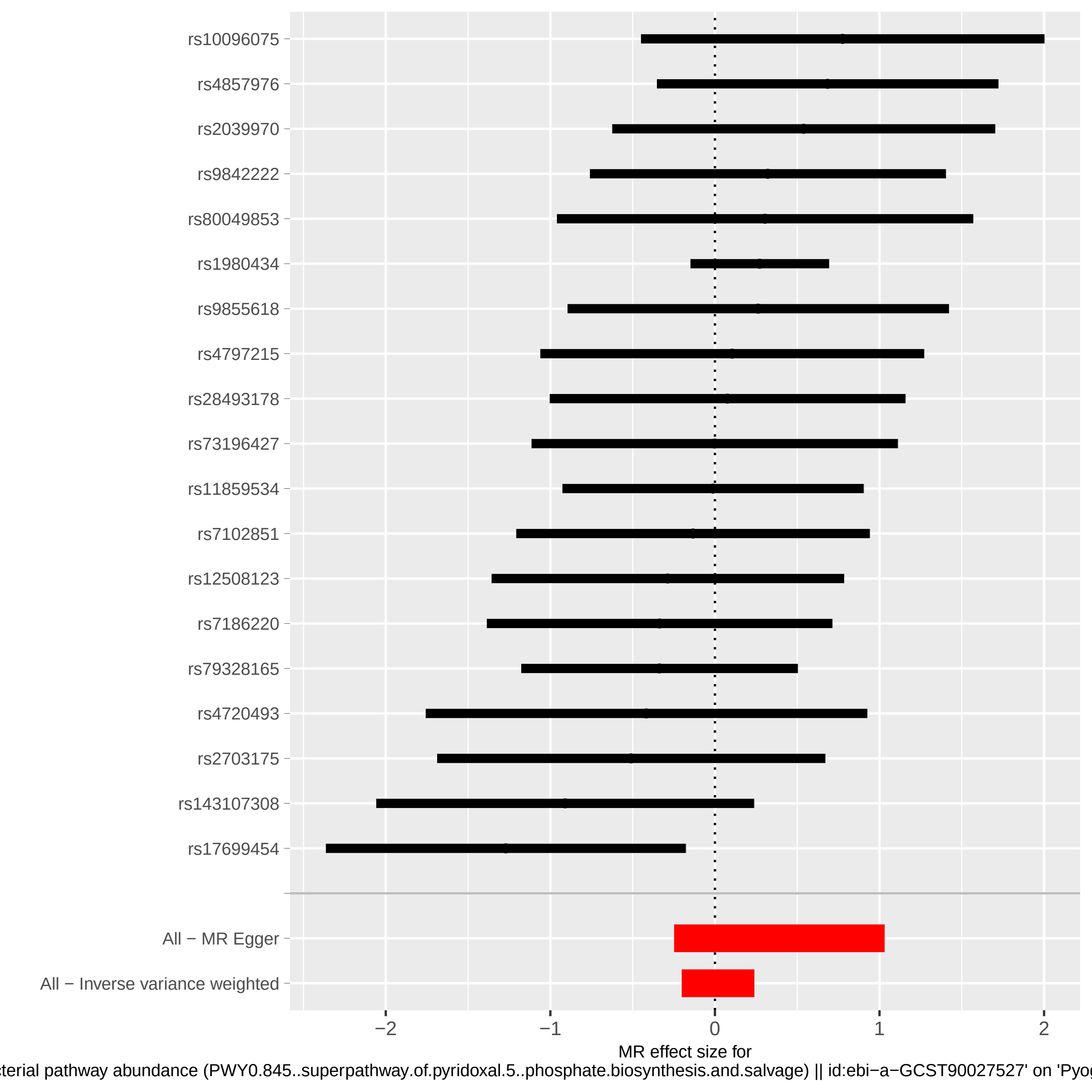
rs10096075
rs4857976
rs2039970
rs9842222
rs80049853
rs1980434
rs9855618
rs4797215
rs28493178
rs73196427
rs11859534
rs7102851
rs12508123
rs7186220
rs79328165
rs4720493
rs2703175
rs143107308
rs17699454
All − MR Egger
All − Inverse variance weighted
−2
−1
0
1
2
MR effect size for
cterial pathway abundance (PWY0.845..superpathway.of.pyridoxal.5..phosphate.biosynthesis.and.salvage) || id:ebi−a−GCST90027527' on 'Pyo
